# Stedman’s Medical Dictionary

**Published:** 1912-11-15

**Authors:** 


### BIBLIOGRAPHY.

STEDMAN'S MEDICAL DICTIONARY.— A new edition of this very practical dictionary has just came from the press of Wm. Wood & Co. It is a model of the printer art in every way, convenient in size, with many new and helpful illustrations. That a new edition should be required in so short a time is sufficient evidence of the worth of the work and the need for it. Dr. Stedman's active touch with modern medical literature makes him peculiarly fitted to supply the wants of the active worker in medical science and practice. The book bears evidence of considerable revision, over two thousand new words or corrections have been made and this work is now unquestionably up to date. No better dictionary could be placed in your dental library.

